# Angiogenesis Inhibitor Bevacizumab Increases the Risk of Ischemic Heart Disease Associated with Chemotherapy: A Meta-Analysis

**DOI:** 10.1371/journal.pone.0066721

**Published:** 2013-06-20

**Authors:** Xing-Lin Chen, Ying-Hong Lei, Cun-Fei Liu, Qun-Fang Yang, Pei-Yuan Zuo, Cheng-Yun Liu, Chang-Zhong Chen, Yu-Wei Liu

**Affiliations:** 1 Key Laboratory of Geriatrics of Health Ministry, Department of Geriatrics, Union Hospital, Tongji Medical College, Huazhong University of Science and Technology, Wuhan, China; 2 Department of Geriatrics, Renji Hospital, Shanghai Jiao Tong University School of Medicine, Shanghai, China; 3 Department of Hyperbaric Oxygen Center, Union Hospital, Tongji Medical College, Huazhong University of Science and Technology, Wuhan, China; 4 Microarray Core Facility, Dana-Farber Cancer Institute, Boston, Massachusetts, United States of America; Universidade Federal do Rio de Janeiro, Brazil

## Abstract

Concerns have arisen regarding the risk of ischemic heart disease with the novel antiangiogenic agent bevacizumab, a recombinant humanised monoclonal antibody to the vascular endothelial growth factor that is widely used in cancer treatment. Currently, the role of bevacizumab in ischemic heart disease is controversial. This meta-analysis was therefore performed to assess the overall risk of ischemic heart disease associated with the use of bevacizumab. The databases of PubMed, EMBASE and Web of Science were searched for English language studies of randomised controlled trials comparing bevacizumab with control therapy published through October 25, 2012. Summary incidence rates, relative risks (RRs) and 95% confidence intervals (CIs) were calculated using random-effects or fixed-effects models based on the heterogeneity of the included studies. A total of 4,617 patients from 7 randomised controlled trials were identified and included for analysis. Among those patients receiving bevacizumab, the summary incidence of ischemic heart disease was 1.0% (95% CI, 0.6%–1.4%). Patients treated with bevacizumab had a significantly increased risk of ischemic heart disease with an RR of 2.49 (95% CI, 1.37–4.52) compared with controls. In addition, both high doses and low doses of bevacizumab increased the risk of cardiac ischemia (low dose at 2.5 mg/kg per week: RR, 2.14 [95% CI, 1.09–4.19]; high dose at 5 mg/kg per week: RR, 4.81 [95% CI, 1.03–22.42]). Bevacizumab was also found to significantly increase the risk of cardiac ischemia in patients with colorectal cancer (RR, 2.13; 95% CI, 1.11–4.06) compared with controls. This meta-analysis shows the use of bevacizumab was associated with an increased risk of developing ischemic heart disease in colorectal cancer patients receiving this drug. Our conclusions are limited by the available data. Further evaluations of high-quality RCTs are needed.

## Introduction

Angiogenesis, a process involving the proliferation of new blood vessels, plays a crucial role in the growth and metastasis of cancer [1]. This process is mainly driven by the vascular endothelial growth factor (VEGF), whose signaling pathway has been a target of many new antineoplastic agents including bevacizumab, sorafenib, sunitinib and others [2], [3]. Bevacizumab (Avastin, Genentech Inc., South San Francisco, California), is a recombinant humanized monoclonal IgG1 antibody that binds to human vascular endothelial growth factor (VEGF) and prevents the interaction of VEGF with its receptors (Flt-1 and KDR) on the surface of endothelial cells. The result is inhibition of endothelial cell proliferation and new blood vessel formation [4]–[6]. It has shown benefits in the treatment of many types of malignancy including colorectal cancer, renal cell carcinoma and breast cancer.

Earlier studies have established the effectiveness of bevacizumab for patients with metastatic carcinoma of the colon or rectum in combination with intravenous fluorouracil-based chemotherapy [7]–[11]; it was also found that bevacizumab prolonged progression-free survival and overall survival and increased one-year survival rates in cancer patients as compared with control therapy [12]. Based on these significant clinical benefits, bevacizumab is currently approved by the Federal and Drug Administration for treatment of advanced colorectal cancer (CRC) (in combination with 5-fluorouracil-based chemotherapy), advanced non-small-cell lung cancer (NSCLC) (in combination with carboplatin and paclitaxel chemotherapy), advanced breast cancer (in combination with paclitaxel chemotherapy), glioblastoma (as a single agent) and metastatic renal cell carcinoma (RCC) (in combination with interferon-a).

Serious adverse effects are associated with the use of bevacizumab, including gastrointestinal tract perforation, wound dehiscence, hemorrhaging, arterial thromboembolic events, hypertensive crises, reversible posterior leukoencephalopathy syndrome, neutropenia and infection, nephrotic syndrome, and congestive heart failure [13]. While bevacizumab is known to be associated with an increased risk of cardiac ischemia [14], it is unclear whether bevacizumab contributes to the development of ischemic heart disease, a common complication leading to morbidity and mortality in patients with malignancy.

Reported incidences of ischemic heart disease in patients treated with bevacizumab varied substantially from 0.52% to 1.7% across phase 3 randomised controlled trials (RCTs) [7], [15]. A recent clinical trial that including 2,526 patients with stage IV colorectal cancer showed that the addition of bevacizumab to cytotoxic combination chemotherapy was associated with a small improvement in overall survival as well as an increased risk of stroke and perforation, but not cardiac events [16]. This result has propagated the view that ischemic heart disease is not one of the more serious adverse effects attributable to bevacizumab. Because the number of patients included in this analysis is limited, the contribution of bevacizumab to ischemic heart disease remains poorly defined.

Numerous other RCTs have been performed over the past years. Several studies heave revealed a higher incidence of ischemic heart disease that is consistently associated with bevacizumab treatment even though it is not significantly different when compared with controls [15], [17].

We hypothesised that the sample sizes in these studies were not large enough to reveal a significantly increased risk of ischemic heart disease due to bevacizumab; therefore, we included published RCTs in a meta-analysis to evaluate the effect of bevacizumab on the occurrence of ischemic heart disease in cancer patients.

## Methods

### Data Sources and Searches

Two investigators searched PubMed, EMBASE, and Web of Science databases for relevant articles published until October 25, 2012; no lower date limit was applied. A sensitive search strategy was performed through terms related to ischemic heart disease, bevacizumab, and randomised trials in all fields. We used the following Medical Subject Heading terms and keywords: “bevacizumab”, “Avastin”, and “carcinoma/cancer”. The search strategy also used text terms such as “ischemic heart disease”, “coronary heart disease”, “myocardial infarction”, “unstable angina”, “coronary artery disease”, “angina pectoris” and “vascular endothelial growth factor” to identify relevant information. We entered Boolean operators (AND, OR, NOT) to combine or exclude search terms. The search was limited initially to English publications of RCTs in humans. We screened the reference lists of included studies and related publications. The results were then hand searched for eligible trials. Results were double-checked and arbitrated by a second investigator.

### Study Selection

The primary goal of this study was to determine whether bevacizumab contributes to the development of ischemic heart disease in cancer patients. Therefore, we selected only those RCTs that directly compared patients with cancer treated with and without bevacizumab. Phase I and single-arm phase II trials were excluded due to their lack of control groups. Specifically, clinical trials that met the following criteria were included in the meta-analysis: prospective phase II and III RCTs in patients with cancer; random assignment of participants to bevacizumab treatment or control (placebo or best supportive care) in addition to concurrent chemotherapy and/or biological agent. We excluded studies that were not published as full reports, such as conference abstracts and letters to editors.

### Data Extraction and Quality Assessment

To avoid bias in the data-abstraction process, 2 investigators independently abstracted the data from the trials and subsequently compared the results. We extracted details about the number of patients, type of cancer being treated, treatment information, follow-up, and funding or support from the included studies. The adverse event was ischemic heart disease (myocardial infarction, unstable angina, coronary revascularization, coronary artery disease, arrhythmias, sudden death or cardiovascular-related death) reported by the investigators during the period of the trial. Data regarding the occurrence of ischemic heart disease was obtained from the safety profile of each study. Two authors (X.L.C. and P.Y.Z.) extracted the data independently, and any discrepancies between the authors were resolved by consensus. When studies compared 2 doses of bevacizumab with a control, we used data from both 2 doses groups. All data were checked for internal consistency, and disagreements were resolved by discussion among the investigators. Quality was assessed using criteria including adequate blinding of randomization, completeness of follow-up, and objectivity of outcome measurements as described previously [18].

### Statistical Analysis

All statistical analysis was implemented with STATA 11.0 (STATA Corp, College Station, Texas) and RevMan 5 (http://ims.cochrane.org/revman/download). All p-values were two-sided. We calculated the incidence by using the number of patients with ischemic heart disease and total number of patients receiving bevacizumab. The proportion of the patients with ischemic heart disease and exact 95% CI were derived from each study. To calculate relative risk (RR), patients assigned to bevacizumab were compared only with those assigned to the control group in the same clinical trial. We explored a dose-effect relationship by further dividing bevacizumab therapy into low dose (5 or 7.5 mg/kg per dose per schedule, which is equivalent to 2.5 mg/kg per week) and high dose (10 or 15 mg/kg per dose per schedule, which is equivalent to 5 mg/kg per week). The low-dose and high-dose designation was arbitrary.

For the meta-analysis, we used a fixed-effects (weighted with inverse variance) or random-effects model based on the heterogeneity of included studies [19]. For each meta-analysis, the Cochran’s *Q* statistic and *I* ^2^ statistics were first calculated to assess the heterogeneity among the proportions of the included trials. If the p-value was less than 0.10, the assumption of homogeneity was deemed invalid, and the random-effects model was reported after exploring the causes of heterogeneity [20]. When results of the two models were substantially different, the random-effects model was presented. Otherwise, the fixed-effects model was reported.We used Begg’s and Egger’s tests [21] to detect possible publication bias.

## Results

### Eligible RCTs

**Figure 1:**
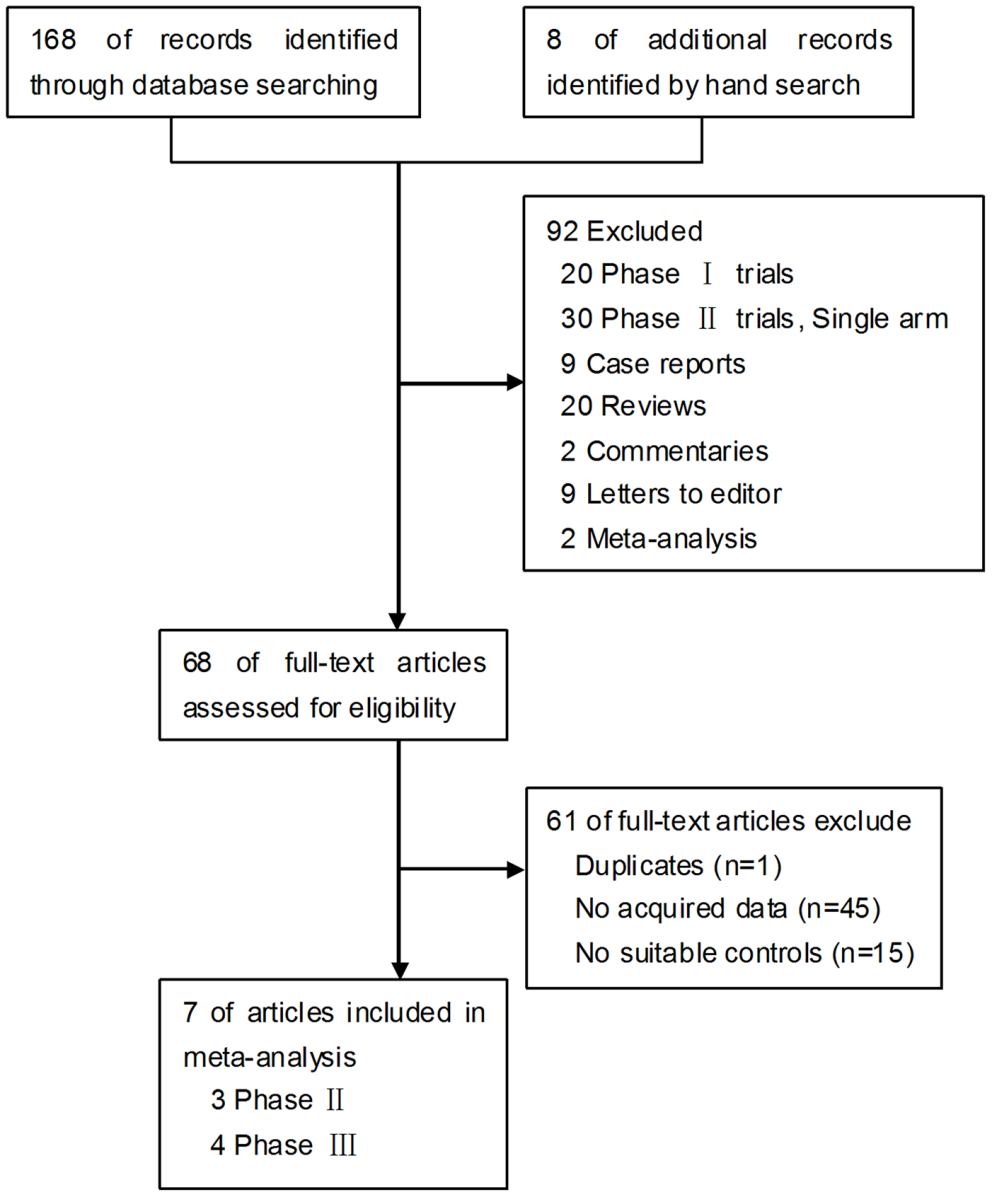
A flow chart showing the progress of trials through the review.

Our search yielded a total of 168 potentially relevant clinical studies on bevacizumab (see Figure 1 for the selection process of these studies). We excluded review articles, case reports, meta-analysis, single-arm phase 2 studies, and RCTs with both arms containing bevacizumab. After independent review, sixty-eight publications reporting RCT results with bevacizumab in patients with various cancers were considered to be eligible for inclusion in the analysis. Of 68 publications, 1 was excluded because the same authors published several reports on the same patients, and only the best-quality study was considered, 45 were excluded because they did not provide acquired data for calculating OR values, 15 were excluded because they did not include suitable control groups. Subsequently, 7 publications [7], [15], [17], [22]–[25] (3 phase II and 4 phase III trials) were available for analyzing the effect of bevacizumab on the risk of ischemic heart disease in patients with cancer. This meta-analyses fully complies with the PRISMA Statement (see Checklist S1) for systematic reviews and meta-analyses.

### Study Characteristics and Quality

Baseline characteristics of the 7 RCTs included in the present meta-analysis are listed in Table 1. These RCTs were all published since 2003. Five RCTs were from USA, 1 from Australia, 1 from Germany. The quality of all the trials was acceptable. No evidence of publication bias was detected for the primary endpoint of this study (relative risk of cardiac ischemia) by either Begg’s or Egger’s test (Begg’s test, p = 0.296; Egger’s test, p = 0.551).

**Table 1:** Characteristics of randomized controlled trials included in the meta-analysis.

Study Name	Trialphase	No.Enrolled	No. forAnalysis	Duration of Follow-up,Median (range), mo	Underyingmalignancy	Concurrent Treatment	Ischemic heart disease	Bevacizumab Dose,mg/kg per wk (b)
Allegra et al. [23] 2009	3	2710	2647	28.5 (NA)	Colorectal Cancer	Fluorouracil, Leucovorin, and Oxaliplatin	Cardic ischemic	2.5
Giantonio et al. [7] 2007	3	829	572	28 (NA)	Colorectal Cancer	Fluorouracil, Leucovorin, and Oxaliplatin	Cardic ischemic	5
Kemeny et al. [17] 2011	2	56	56	NA	Liver Cancer	Floxuridine, and Dexamethasone	Myocardial infarct	5
Moehler et al. [24] 2009	2	46	46	19.5 or 17.0* (NA)	Colorectal Cancer	Capecitabine, and Irinotecan	Cardiovascular	2.5
Price et al. [15] 2012	3	471	471	30.8 (NA)	Colorectal Cancer	Capecitabine, and Mitomycin C	Cardiac ischaemia/infarction/angina	2.5
Rini et al. [22] 2010	3	732	709	NA	Renal cell Carcinoma	Interferon alfa	Cardiac ischemia/infarction	5
Yang et al. [25] 2003	2	116	116	27(NA)	Renal cell Carcinoma	None	Cardic ischemic	1.5 or 5

Abbreviations and notes: NA, data not available.

* The resulting median follow-up times for CAPIRI and CAPIRI-Bev were similar, 19.5 and 17.0 mo, respectively.

(a) Funding sources: Three trials were supported by National Cancer Institute [7], [17], [22], [23]. One trial was sponsored by Genentech [17]. One trial was sponsored by Pfizer and Roche [24]. One trial was sponsored by Roche Australia [15].

(b) The dose schedule was converted from mg/kg per schedule.

### Incidences of Cardiac Ischemia

A total of 4,617 patients from 7 randomised controlled trials were identified and included for analysis. Among those patients receiving bevacizumab, the summary incidence of ischemic heart disease was 1.0% (95% CI, 0.6%–1.4%), using a random-effects model (see Table 2).

**Table 2:** Incidence and relative risk (RR) of cardiac ischemia with bevacizumab according to tumor types.

	No. ofStudies	Cardiac ischemia (No./Total N0.) Bevacizumab	Cardiac ischemia (No./Total N0.) Control	Incidence(95% CI), %	RR(95% CI)
Overall	7	41/2417	13/2200	1.0 (0.6–1.4)	2.49 (1.37–4.52)
Colorectal Cancer	4	33/1957	13/1779	1.1 (0.5–1.8)	2.13 (1.11–4.06)
Renal cell Carcinoma	2	7/438	0/387	0.8 (0.2–1.4)	6.12 (0.83–45.43)
Liver Cancer	1	1/22	0/34	–	4.57 (0.19–107.29)

Abbreviation and notes: CI, confidence interval. The incidence and RR were calculated from the trials included in this study by meta-analysis as described in the “Method” section.

### Relative Risk of Cardiac Ischemia

The observed incidence of cardiac ischemia associated with bevacizumab may be attributable to known or potential risk factors such as hypertension, diabetes, hyperlipidemia, chemotherapy, or malignancy. In order to determine the particular contribution of bevacizumab to the occurrence of cardiac ischemia, and to exclude the influence of confounding factors, we calculated overall relative risk of cardiac ischemia from these RCTs, in which a direct comparison was made between bevacizumab and a control with concurrent standard chemotherapy therapy.

**Figure 2:**
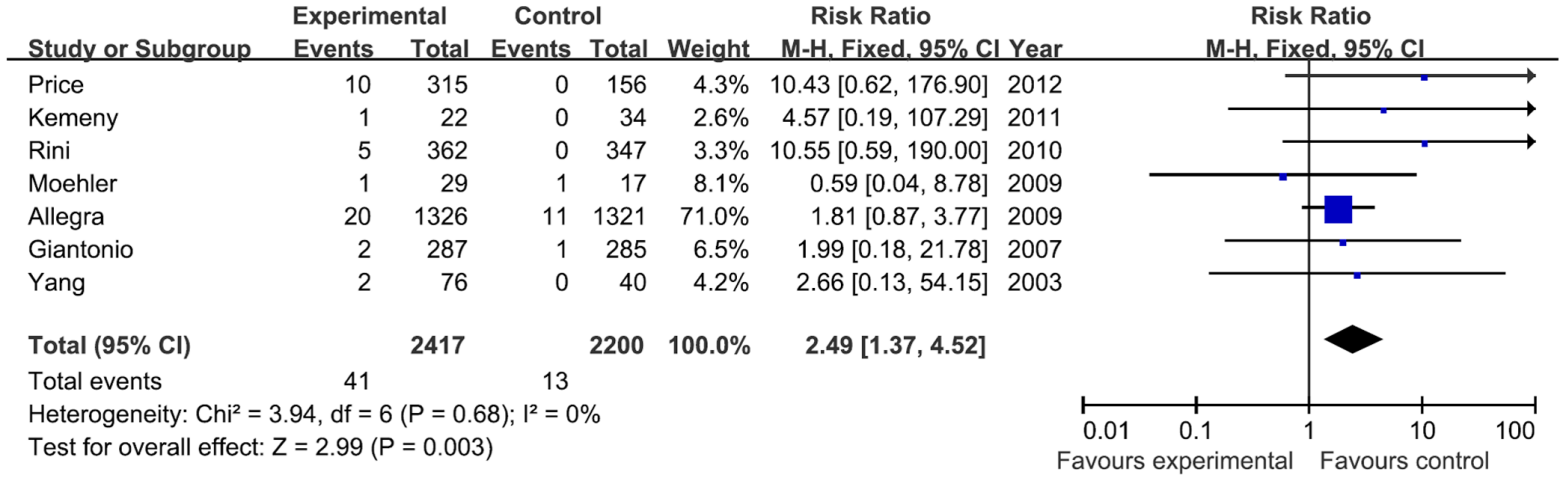
Meta-analysis of the relative risk (RR) of cardiac ischemia between bevacizumab and control therapy using fixed-effects model. **Bars, 95% confidence intervals (CI) of RR in patients receiving bevacizumab versus controls.** The areas of the squares are proportional to the weights used for combining the data. The center of the lozenge gives the combined RR. The RR was considered statistically significant if the 95% CI for the overall RR did not overlap one.

We performed a meta-analysis to calculate the overall RR associated with bevacizumab in comparison with controls. No heterogeneity was found among these studies included in the analysis despite clear disparity in tumor type and related treatment (Figure 2). Using a fixed-effect model, the summary overall RR for bevacizumab versus control was 2.49 (95% CI, 1.37–4.52). Thus, there was a significantly increased risk of cardiac ischemia in patients treated with bevacizumab. The overall risk of cardiac ischemia in patients receiving bevacizumab was 149% greater than control treatment.

### Subgroup Analyses

#### Infuence of bevacizumab dose on the risk of cardiac ischemia

**Figure 3:**
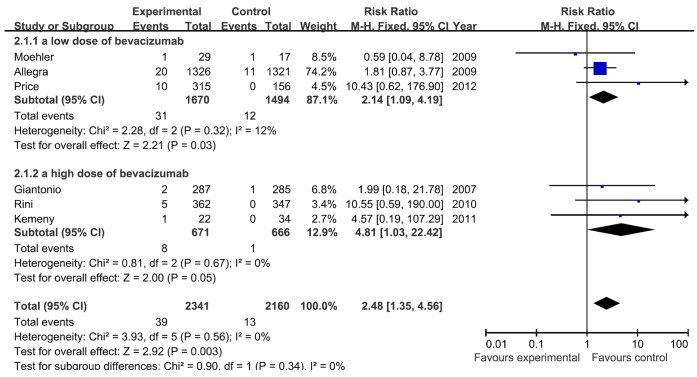
Meta-analysis of the relative risk (RR) of cardiac ischemia associated with bevacizumab at 2.5 or 5 mg/kg/week when compared to controls using fixed-effects model. Bars, 95% confidence intervals (CI) of RR in patients receiving bevacizumab versus controls. The areas of the squares are proportional to the weights used for combining the data. The center of the lozenge gives the combined RR. The RR was considered statistically significant if the 95% CI for the overall RR did not overlap one.

We explored the relationship between the dose of bevacizumab and RR of cardiac ischemia to further understand the role of bevacizumab in the development of cardiac ischemia. A meta-analysis was performed to calculate the relative risk associated with bevacizumab at 2.5 or 5 mg/kg/week when compared to controls (Figure 3). The RR of cardiac ischemia for bevacizumab at 2.5 mg/kg/week was 2.14 (95% CI, 1.09–4.19) from 3 RCTs including 3,164 patients (bevacizumab 1,670, controls 1,494). The RR of cardiac ischemia at 5 mg/kg/week was 4.81 (95% CI, 1.03–22.42) from 3 RCTs including 1,337 patients (bevacizumab 671, controls 666). Therefore, both high doses and low doses of bevacizumab increased the risk of cardiac ischemia. The interaction test shows no statistical difference between these subgroups (I^2^ = 0%; p = 0.34).

#### Risk of cardiac ischemia and tumor type

In order to investigate the relationship between cardiac ischemia and tumor type, we determined the risk of cardiac ischemia with bevacizumab according to tumor histology. The incidence and RR of cardiac ischemia varied with different tumors (Table 2). The incidence of cardiac ischemia in patients with colorectal cancer was 1.1% (95% CI, 0.5%–1.8%). Bevacizumab was also found to significantly increase the risk of cardiac ischemia in patients with colorectal cancer (RR, 2.13; 95% CI, 1.11–4.06) compared with controls (Table 2). But bevacizumab was not found to significantly increase the risk of cardiac ischemia in patients with renal cell carcinoma or liver cancer in comparison with controls (RR, 6.12; 95% CI, 0.83–45.34; RR, 4.57; 95% CI, 0.19–107.29). The interaction test shows no statistical difference between these subgroups (I^2^ = 0%; p = 0.57).

## Discussion

Cancer treatment today employs a combination of chemotherapy, radiotherapy, and surgery to prolong life and provide cure. However, many of these treatments can cause cardiovascular complications such as heart failure, myocardial ischemia/infarction, hypertension, thromboembolism, and arrhythmias [26]–[28]. The risk of hypertension is increased three times with low dose and 7.5 times with high dose of bevacizumab [29]. About 2–4% of patients (mostly pretreated with anthracyclines or irradiated to mediastinum) develop CHF, and 4–5%-arterial thromboembolic events. The use of bevacizumab is related to doubled risk of stroke, myocardial infarction, coronary disease and cardiac death [27], [30]. There have been postmarketing reports of adverse cardiac ischemic events associated with the use of bevacizumab [7], [15]. To improve the diagnosis and management of the cardiovascular side effects of anti-angiogenic drugs, the dialogue between oncologists and cardiologists should be strengthened [31]. The association of ischemic heart disease with new agents presents a challenge for recognition because many RCTs may not be powered to reveal a significant relationship. Our meta-analysis of 7 RCTs has overcome this limitation of individual trials and demonstrated that bevacizumab may be associated with a significantly increased risk of ischemic heart disease (RR, 2.49; 95% CI 1.37–4.52) in patients with 3 kinds of tumors. This finding will help physicians and patients to recognize the risk of ischemic heart disease with the administration of bevacizumab.

The risk of bevacizumab-associated ischemic heart disease may be underestimated by previous studies, which showed no increased risk of ischemic heart disease with bevacizumab compared with a control [16]. The failure to detect such an increase in ischemic heart disease risk is likely due to the limited number of trials included for the analysis. Because bevacizumab is increasingly used in the routine treatment of cancer patients and in the setting of clinical trials in combination with other agents [32]–[35], it is important for oncologists and primary care physicians to be aware of the increased incidence of ischemic heart disease associated with bevacizumab to monitor and treat it appropriately [36].

The mechanism behind bevacizumab increasing the risk of ischemic heart disease has been mostly linked to a pathologic perturbation at the level of the endothelial cell mediated by VEGF depletion. VEGF is also known to increase nitric oxide (NO) production by endothelial cells with resulting antiplatelet actions and inhibition of leukocyte adhesion [37], [38]. Inhibition of VEGF may increase the risk of cardiac ischemic events. The endothelial cell plays a critical role in vascular homeostasis and VEGF provides a vascular protective effect on the endothelial cell effect through anti-apoptosis, anti-inflammation and survival [39]. Bevacizumab may increase expression of proinflammatory cytokines causing damage in ischemic heart. The most serious, and sometimes fatal, bevacizumab toxicities are gastrointestinal perforation, wound-healing complications, hemorrhage, arterial thromboembolic events, hypertensive crisis, nephrotic syndrome and congestive heart failure [40]. Furthermore, a recent meta-analysis that including 3060 patients with colorectal cancer has showed that the addition of bevacizumab was related to increased rates of hypertension, proteinuria, bleeding and thromboembolic events, also leading to a slight increment on treatment interruptions. Other variables, such as hematologic toxicity and gastrointestinal perforation, were not statistically significant [41].

Our study showed that the summary incidence of ischemic heart disease was 1.0% (95% CI, 0.6%–1.4%) and the RR was 2.49 (95% CI, 1.37–4.52) in patients with solid tumors who had been treated with bevacizumab. It is evident that risks of ischemic heart disease are all substantial among bevacizumab. Our results are consistent with those addressed in the review published in 2011 [30]. Many factors such as age, functional status, stage and histology of the malignant tumor, mobility, concurrent chemotherapy and prothrombotic states are known to contribute to the development of ischemic heart disease in cancer patients. We also explored risk factors for ischemic heart disease associated with bevacizumab. Our study demonstrated that the incidence of ischemic heart disease with bevacizumab varies significantly among patients with different types of tumors; higher risk was associated with colorectal cancer. But bevacizumab was not found to significantly increase the risk of cardiac ischemia in patients with renal cell carcinoma or liver cancer in comparison with controls. The difference in ischemic heart disease by cancer type also may reflect the extent of prior treatment and performance status in addition to its biology. The high risk of ischemic heart disease associated with colorectal cancer suggests a need for prophylaxis in these patients when treated with bevacizumab.

In addition to tumor type, another potential risk factor for ischemic heart disease may be the dose of bevacizumab. A high dose of bevacizumab (5 mg/kg per week) was found to be associated with a significantly increased risk of ischemic heart disease with an RR of 4.89 (95% CI, 1.04–23.04) in comparison with controls. However, for patients who were treated with a low dose of bevacizumab (2.5 mg/kg per week), their risk of ischemic heart disease also was significantly increased (RR, 2.14; 95% CI, 1.09–4.19). The interaction test did not showed significant differences in the cardiac risk between high dose and low dose subgroups (I^2^ = 0%; p = 0.34). Apparently, even low-dose bevacizumab is associated with the increased risk of ischemic heart disease. The data suggests that the so called low dose of bevacizumab may be already reaching the saturation level to induce cardiac ischemia.

Our study has the following limitations. First, the ability to detect ischemic heart disease may vary among institutions in which these trials were performed, and may cause bias of the reported incidence rates. A higher risk of adverse events was observed in patients with colorectal cancer - probably the statistical difference found on RR was related only to the sample size. Because in all groups of patients treated with bevacizumab the risk of ischemic heart disease was about 1%. As the number of patients included in this analysis was limited, the contribution of bevacizumab to ischemic heart disease in colorectal cancer patients remain poorly defined. There are two hypotheses to explain this result: firstly, the fact that trials being as divergent in their results as in their designs (number of patients, selection criteria, bevacizumab dose) could influence the data and consequently introduce bias. The second explanation is a possible interaction of bevacizumab. Second, the incidences of ischemic heart disease showed significant heterogeneity among the included studies. This may reflect differences in sample sizes, tumor type, concomitant chemotherapies, and many other factors among these studies. Despite these differences, the RRs reported by all of these studies showed remarkable nonheterogeneity. In addition, calculation using the random-effects model for incidence estimation may be able to minimize the problem. Third, the present study has the typical limitations of the meta-analytical methodology. Our findings and interpretations were limited by the quality and quantity of data that is available. An analysis of individual patient data would be more powerful to confirm our findings. The search covered a range of relevant sources. However, it was restricted only to English publications. Another concern is the possible existence of some unpublished studies, which could lead to potential publication bias, although we found no indication of such bias by using statistical methods designed to detect it. And we thought it was essential to evaluate the risk of cardiac events related to the combination of bevacizumab with different chemotherapy regimens. Our results indicated that bevacizumab was not found to significantly increase the risk of cardiac ischemia in 5-FU regimens patients in comparison with controls (OR, 1.92; 95% CI, 0.97–3.83). Future studies can focus on the separate comparison of ischemic heart disease associated with bevacizumab, according to cytotoxic elements involved and pattern of 5-FU administration. Finally, this is a meta analysis at the study level, and confounding factors at the patient level cannot be properly assessed and incorporated into the analysis.

In conclusion, our study has shown that the novel antiangiogenic agent bevacizumab is associated with a significantly increased risk of ischemic heart disease in colorectal cancer patients who receive concurrent chemotherapy or cytokine therapy. The risk is increased with both high and low doses of bevacizumab. The RR of ischemic heart disease may vary with tumor type. It is imperative for physicians and patients to recognize the risk. Our conclusions are limited by the available data. Bevacizumab may be continued if benefits of the drug outweigh the risk. Future studies are needed to investigate the prevention and management of ischemic heart disease associated with bevacizumab, especially in breast cancer, lung cancer, and ovarian cancer patients and its dose are important issues that need further evaluation by high-quality RCTs. Also, the need for detailed description of adverse events, especially in ischemic heart disease, in primary studies should be enhanced.

## Supporting Information

Checklist S1 PRISMA 2009.
(DOC)
